# Polyphenols, Polysaccharides, and Their Complexes from *Aronia melanocarpa* in the Chemoprevention of Colorectal Cancer

**DOI:** 10.3390/molecules31010010

**Published:** 2025-12-19

**Authors:** Karolina Niewinna, Katarzyna Owczarek, Zuzanna Senkowska, Urszula Lewandowska

**Affiliations:** Department of Biochemistry, Faculty of Medicine, Medical University of Lodz, 90-419 Lodz, Poland; karolina.niewinna@umed.lodz.pl (K.N.); katarzyna.owczarek@umed.lodz.pl (K.O.); zuzanna.senkowska@student.umed.lodz.pl (Z.S.)

**Keywords:** *Aronia melanocarpa*, blackberry, bioavailability, inflammation, chemoprevention, CRC, polyphenols, polysaccharides

## Abstract

Colorectal cancer (CRC) is among the three most commonly diagnosed malignancies worldwide and remains a major public health challenge, emphasizing the need for effective preventive strategies. Considering the current chemotherapy limitations of key agents, natural products widely researched as dietary supplements can complement conventional treatments. This review concentrates on *Aronia melanocarpa* (black chokeberry), including its fruits, leaves and pomace, as a rich source of bioactive compounds with well-documented anticancer properties. Notably, *A. melanocarpa* contains high levels of polyphenols such as cyanidin-3-galactoside, cyanidin-3-arabinoside, chlorogenic acid, quercetin, and epicatechin, as well as biologically active polysaccharides, including pectins and arabinogalactans. These compounds, through their antioxidant and anti-inflammatory activities, are involved in modulating apoptosis pathways specifically targeting cancer cells. Moreover, their complexes may enhance chemopreventive efficacy through synergistic mechanisms. Recent studies show that supplementation with aronia products can improve inflammatory markers such as interleukin-6 and tumor necrosis factor alpha, highlighting its potential role in modulating the tumor microenvironment. Collectively, these findings position *A. melanocarpa* as a promising candidate for use in integrative strategies aimed at the prevention and adjunctive treatment of CRC.

## 1. Introduction

### 1.1. Overview of Colorectal Cancer

Colorectal cancer (CRC) is one of the most commonly diagnosed malignancies and a leading cause of cancer-related mortality worldwide, with 1.93 million new cases and 935,000 deaths reported in 2020 [1]. While incidence remains highest in high-income countries, rising rates in low- and middle-income regions are linked to Westernized lifestyles. It should be emphasized that, based on projections related to aging, population growth, and societal development, the annual number of newly diagnosed CRC cases is expected to reach approximately 3.2 million by 2040, with annual deaths rising to about 1.6 million. The anticipated increase in CRC incidence is primarily associated with greater exposure to environmental risk factors [2].

Modifiable risk factors such as diets rich in red and processed foods, low fiber intake, obesity, physical inactivity, alcohol consumption or smoking significantly contribute to CRC development [3]. A recent study confirms that pro-inflammatory lifestyle patterns strongly correlate with increased CRC cases [4]. Chronic inflammatory bowel diseases (IBD), especially when associated with primary sclerosing cholangitis, further elevate CRC risk due to persistent mucosal inflammation [5]. Advances in molecular diagnostics and endoscopic surveillance have improved CRC risk stratification in IBD populations [6]. Factors such as age, sex, family history, and hereditary syndromes (e.g., Lynch syndrome, familial adenomatous polyposis) also play a critical role [7]. CRC is largely preventable through early detection and screening, though access imbalance carry on globally [8]. At present, considerable emphasis is placed on the prevention of CRC development, with particular focus on chemoprevention, as this approach has the potential to substantially reduce disease incidence. This strategy involves the search for agents—primarily of natural origin—to inhibit, delay, or reverse carcinogenesis, and has shown promise in high-risk populations as a complementary approach alongside screening and lifestyle interventions.

### 1.2. The Role of Chemoprevention in CRC

Chemoprevention is a rapidly evolving and promising strategy for the prevention of CRC, largely due to the slow progression of the disease from precancerous adenomas to invasive carcinomas. This extended latency provides a crucial opportunity to intervene using natural or synthetic agents capable of halting or delaying tumor development [9]. Among various approaches, dietary chemoprevention has garnered particular interest, supported by the well-established associations between nutrition, lifestyle and CRC risk [10].

Plant-derived bioactive compounds, particularly polyphenols, exert chemopreventive activity through multiple biological mechanisms. These include the modulation of oxidative stress, suppression of chronic inflammation and regulation of key cancer-related signaling pathways [11,12]. Notably, polyphenols such as resveratrol, quercetin, epigallocatechin gallate and curcumin have shown potent antioxidant, anti-inflammatory, pro-apoptotic, and anti-angiogenic properties in CRC studies [10,13].

In addition to direct effects on tumorigenic processes, dietary polyphenols and plant-derived polysaccharides also influence colorectal carcinogenesis via modulation of the gut microbiota. These compounds can enhance epithelial barrier integrity, regulate host immune responses and shape a microbial environment that supports anti-inflammatory and anticancer outcomes [10]. Furthermore, microbial metabolism of dietary components leads to the production of key bioactive metabolites, such as short-chain fatty acids (SCFAs)—notably butyrate, acetate and propionate acids—which have been shown to induce apoptosis, suppress inflammation and regulate gene expression in colonic epithelial cells [14].

Emerging evidence also supports the concept of synergistic interactions among multiple bioactive compounds naturally present in whole food, such as *Aronia melanocarpa*, enhance their chemopreventive potential. This supports the focus on this botanical source as a promising dietary strategy for CRC prevention, particularly in the context of its rising global incidence [10,11,12].

### 1.3. Significance of Aronia melanocarpa in Preventive Medicine

Black chokeberry (*Aronia melanocarpa*) is a plant resident to the Rosaceae family. This particular species is gaining increasing recognition as a functional food with exceptional potential in the prevention of chronic diseases. Its health-promoting effects stem primarily from its remarkably high content of polyphenolic compounds—particularly anthocyanins, proanthocyanidins, flavonoids and phenolic acids—which exhibit strong antioxidant, anti-inflammatory and cytoprotective properties [15]. Compared to other berries and polyphenol-rich fruits, *A. melanocarpa* demonstrates superior capacity to modulate pathways relevant to colorectal cancer (CRC) prevention, including oxidative stress, NF-κB-mediated inflammation, gut microbiota composition, and intestinal barrier integrity [15,16,17].

In vitro studies have demonstrated that extracts from chokeberry leaves and berries can induce apoptosis, inhibit proliferation, and suppress migration and invasiveness of cancer cells by modulating pathways such as NF-κB, epithelial–mesenchymal transition (EMT), and angiogenesis [18,19]. Different studies also highlight chokeberry polyphenols’ ability to inhibit LPS-induced inflammation in colon epithelial cells and macrophages, further reinforcing their relevance in CRC-related inflammatory pathways [20]. These anticancer effects align with broader polyphenol mechanisms, such as cell cycle arrest, induction of apoptosis, inhibition of angiogenesis, and suppression of tumor-promoting signaling pathways—previously described in comprehensive reviews [21].

In vivo studies indicate that chokeberry supplementation can improve oxidative stress markers, lipid profiles, glucose metabolism, and gut microbiota composition, supporting gastrointestinal health and intestinal barrier integrity [16,17].

Clinical studies in humans, more limited, have reported beneficial effects on metabolic and inflammatory markers, including reductions in C-reactive protein (CRP), improvements in lipid and glucose profiles, and enhanced antioxidant enzyme activity (SOD, GPx, CAT) following juice or extract consumption [22].

Extracts from various aronia species show cytotoxic and antiproliferative activity against several cancer cell types—including colorectal, breast and prostate cancer—highlighting their broad chemopreventive potential [20]. These effects are linked to the high content of polyphenols and their ability to modulate oxidative stress and inflammation. Systematic reviews further confirm that chokeberry supplementation reduces markers of inflammation and oxidative stress while enhancing endogenous antioxidant enzyme activity [23,24].

## 2. Chemical Composition of *Aronia melanocarpa*

*Aronia melanocarpa* possesses a complex and well-defined chemical composition that includes a wide spectrum of bioactive ingredients. Its fruits and pomace are particularly rich in polyphenolic compounds, such as anthocyanins (mainly cyanidin derivatives), proanthocyanidins, flavonols (including quercetin and kaempferol glycosides) and phenolic acids (notably chlorogenic, neochlorogenic and caffeic acids) [15,25,26]. These compounds are found in both free and bound forms and vary in concentration depending on the plant part (leaves, fruit, pomace) and processing method (Table 1).

Besides polyphenols, aronia contains significant levels of polysaccharides, pectins and dietary fiber, which are mostly retained in pomace after juice extraction [40,41]. The fruit matrix also includes organic acids (e.g., malic and citric acids), natural sugars (mainly glucose and fructose) and trace amounts of unsaturated fatty acids. Furthermore, aronia provides essential minerals, such as potassium, magnesium, calcium, phosphorus and iron, as well as small quantities of vitamins, particularly vitamin C and some B-complex vitamins [16].

### 2.1. Polyphenolic Compounds in Aronia melanocarpa: Types and Biological Activity

Polyphenols are a structurally diverse group of secondary plant metabolites known for their antioxidant, anti-inflammatory and antiproliferative activities, particularly relevant in the context of CRC chemoprevention [42]. This category includes subclasses such as flavonoids, phenolic acids, stilbenes, lignans and less commonly in food matrices, curcuminoids [43]. These compounds vary not only in their chemical structures but also in their concentration. *Aronia melanocarpa* is among the richest natural sources of polyphenols, whose presence and biological effects have been extensively documented.

Flavonoids, characterized by a C6-C3-C6 carbon skeleton comprising two phenyl rings connected via a heterocyclic pyran or pyrone ring. They are subclassified based on ring modifications such as hydroxylation, glycosylation, or methylation into flavonols, flavones, flavanols, isoflavones, anthocyanins and flavanones [43].

Flavonols, e.g., quercetin and kaempferol derivatives, occur in *A. melanocarpa* mainly as quercetin-3-*O*-glucoside, quercetin-3-galactoside, quercetin-3-rutinoside, quercetin-3-robinobioside, quercetin-3-vicianoside, isorhamnetin-3-*O*-glucoside and kaempferol-3-*O*-glucoside [23,44]. Concentrations in fruits range from 0.12 to 1.4 mg/g dry weight (DW), while in leaves they may reach 1.8–7.86 mg/g DW [16,27,36]. In addition, comparative studies of leaf extracts from aronia and other polyphenol-rich species have confirmed the presence of high levels of flavonoids, phenolic acids and ellagitannins, correlating with notable antibacterial activity against Gram-positive pathogens [45]. These flavonols have been implicated in modulation of signaling cascades such as Phospho Inositide 3-Kinase/Protein kinase B (PI3K/Akt) and p53, contributing to cell cycle arrest and apoptosis in CRC models. Additionally, quercetin has been shown to enhance the effectiveness of chemotherapeutic agents by inhibiting multidrug resistance transporters, including P-glycoprotein, through both decreased transporter function and reduced expression in various cancer cell models [46].

Flavanols (flavan-3-ols), including monomers such as (+)-catechin and (−)-epicatechin and their polymers (proanthocyanidins), are abundant in *A. melanocarpa*. The dominant polymeric procyanidins account for a substantial portion of the antioxidant capacity (e.g., ~66% of total polyphenols in fruits). Total flavanol content in chokeberry fruits can vary broadly; for example, polymeric procyanidins (mainly epicatechin units) dominate, with free epicatechin in lower amounts [16]. They exert strong radical-scavenging and metal-chelating effects, inhibit NF-κB signaling and downregulate pro-inflammatory cytokines (e.g., IL-6, TNF-α) [20]. Flavanols may also beneficially modulate gut microbiota composition and enhance SCFAs production [14].

Anthocyanins are water-soluble pigments responsible for the deep color of chokeberries. In *Aronia melanocarpa*, anthocyanins are the predominant polyphenolic group, accounting for up to 60% of the total phenolic content [17,44]. The main anthocyanins are cyanidin derivatives, particularly cyanidin-3-galactoside, also cyanidin-3-arabinose, cyanidin-3-glucoside and cyanidin-3-xyloside. In some studies, cyanidin-3-*O*-galactoside extract of aronia berries reach 917.31 mg/g DW total anthocyanins, after purification [47]. These compounds exhibit potent antioxidant activity, regulate inflammatory cytokines such as TNF-α, IL-6 and have been shown to induce apoptosis in CRC cell lines [48].

Phenolic acids, a major class of non-flavonoid polyphenols are structurally characterized by hydroxyl and carboxyl functional groups and are subdivided into two main categories: hydroxybenzoic acids and hydroxycinnamic acids [43]. Black chokeberry is particularly notable for its high phenolic acid content, with chlorogenic acid and neochlorogenic acid being the dominant representatives. Chlorogenic acid concentrations range from 0.8 to 1.5 mg per g fresh weight (FW), while neochlorogenic acid is typically present at levels of 0.4 to 0.7 mg/g FW [16]. These hydroxycinnamic acids are recognized for their strong antioxidant capacity, primarily through free radical scavenging and metal ion chelation. Furthermore, they exert chemopreventive effects by inducing phase II detoxification enzymes, such as glutathione S-transferase, which contributes to the protection of colonic epithelial cells against oxidative DNA damage and carcinogenesis [49].

Lignans are diphenolic compounds biosynthesized via the shikimate pathway. Upon ingestion, they are metabolized by gut microbiota into enterolignans, such as enterodiol and enterolactone, which exhibit phytoestrogenic, antioxidant and anticancer activities [50]. While *Aronia melanocarpa* is not generally considered a major source of lignans and most compositional studies do not report their presence in significant concentrations in fruit tissues [27], some research suggests that minor lignan components (such as secoisolariciresinol) may occur in small quantities and contribute to the overall antioxidant and anticancer potential of chokeberry extracts [25], but evidence remains limited.

There are also studies comparing the biological activities and synergistic effects of aronia extracts with other polyphenols, such as stilbenes and curcuminoids. Stilbenes are structurally defined by two aromatic rings connected via a two-carbon ethylene bridge, with resveratrol being the most prominent compound, widely recognized for its antioxidant, anti-inflammatory, and cardioprotective properties. Dietary sources of stilbenes include berries [51]. Although *Aronia melanocarpa* is frequently compared to resveratrol in antioxidant and antiplatelet activity studies, current phytochemical analyses do not confirm the presence of resveratrol or other stilbenes in chokeberry fruits in quantifiable amounts [25,52]. In functional assays, chokeberry extracts have shown greater inhibitory effects on platelet aggregation than resveratrol used as a positive control, but this refers to comparative bioactivity rather than endogenous stilbene content [53].

Curcuminoids (e.g., curcumin, demethoxycurcumin, bisdemethoxycurcumin) are a small class of polyphenolic compounds extracted primarily from *Curcuma longa* (turmeric). Curcumin recognized for its potent anti-inflammatory, antioxidant and chemopreventive properties, acting through mechanisms such as NF-κB inhibition and modulation of oxidative stress pathways [54]. These compounds are not naturally present in aronia; however, in nutraceutical formulations or synergy-based studies, chokeberry extracts are often combined with curcuminoids to target overlapping molecular pathways—particularly those involved in inflammation, oxidative stress regulation and tumor suppression—thus enhancing the overall protective effects [25].

Recent clinical evidence also supports the role of polyphenol-based interventions, including curcuminoids, in the prevention and adjunct treatment of CRC. According to López-Gómez and Uranga (2024), combinations of dietary polyphenols such as curcumin with other bioactive compounds can improve treatment outcomes by modulating gut microbiota, reducing pro-inflammatory cytokines and enhancing epithelial barrier integrity—mechanisms that align with those attributed to Black chokeberry [42].

### 2.2. Polysaccharides in Aronia melanocarpa

*Aronia melanocarpa* contains a complex and diverse polysaccharide profile that plays an important role in the nutritional and health-promoting properties of the fruit. The dietary fiber in aronia pomace (the residue after juice extraction) is dominated by cellulose, accounting for approximately 300–350 mg/g DM and hemicelluloses (about 150–200 mg/g DM), including polysaccharides such as arabinogalactans, galactomannans and xyloglucans [34,55]. Pomace also contains significant amounts of pectins, averaging 70–100 mg/g DM, which represent an important soluble fiber fraction [34].

Monosaccharide analysis revealed that the main monosaccharide components of aronia polysaccharides are: arabinose (20–28%), galactose (25–30%), glucose (10–15%), xylose (5–8%), along with smaller amounts of fucose, rhamnose, mannose and galactosamine [34]. The two main polysaccharide fractions obtained from aronia showed average molecular weights of approximately 51,089 Da and 21,214 Da, respectively. Both fractions exhibited low polydispersity index values, indicating that they were molecularly uniform and composed of relatively homogeneous polysaccharide molecules [56].

### 2.3. Synergy Between Polyphenols and Polysaccharides in Natural Products

Recent research has highlighted the importance of synergistic interactions between polyphenols and polysaccharides in natural products, where interactions enhance both the bioavailability and biological efficacy of bioactive compounds. Polyphenols such as anthocyanins, when complexed with polysaccharides like arabinogalactans or xyloglucans, exhibit increased chemical stability and solubility, improving their resistance to gastrointestinal degradation and facilitating intestinal absorption. Wang et al. (2024) demonstrated that anthocyanin-polysaccharide complexes prepared from chokeberry showed significantly enhanced antioxidant activity compared to free anthocyanins, with a 1:1 ratio producing the most favorable structural and functional properties. These complexes formed amorphous, smooth particles, suggesting non-covalent interactions—primarily hydrogen bonding and hydrophobic forces—underlying their stability [55]. In addition to improving antioxidant potential, these complexes influence host health through gut microbiota modulation [57].

Several studies have shown that polyphenol-polysaccharide rich chokeberry extracts promote beneficial microbial shifts, including increased abundance of *Lactobacillus* and *Bifidobacterium* and decreased levels of gut inflammation such as *Sutterella* and *Escherichia* [58]. These microbial changes are accompanied by increased production of SCFAs, such as butyrate, which play key roles in maintaining intestinal barrier function and regulating immune responses. Zhu Y et al. (2022) further demonstrated that chokeberry polyphenols can regulate lipid metabolism by modulating the glycerophospholipid metabolic pathway, offering potential benefits for cardiometabolic health [59]. According to Shahidi and Athiyappan (2025), the functional interplay between polyphenols and polysaccharides may be leveraged in the development of novel food systems and delivery platforms that enhance the therapeutic potential of natural bioactives [60]. Collectively, these findings underscore that the health benefits of natural products should be understood in the context of their molecular synergy, rather than as isolated constituents.

## 3. Action of Polyphenols in Chemoprevention of CRC

### 3.1. Antioxidant and Anti-Inflammatory Properties

Polyphenols extracted from black chokeberry exhibit strong biological properties in the chemoprevention of CRC. One of the key mechanisms is their ability to neutralize reactive oxygen species (ROS), which are known to cause oxidative DNA damage and initiate carcinogenesis. Aronia polyphenols—particularly anthocyanins and proanthocyanidins—act as radical scavengers, chelate pro-oxidant metals and inhibit ROS-producing enzymes such as NADPH oxidase and cyclooxygenase [55,61]. Additionally, they upregulate the Nrf2/ARE pathway, leading to increased expression of endogenous antioxidant enzymes like superoxide dismutase (SOD) and glutathione peroxidase (GPx), thereby reinforcing cellular antioxidant defenses [37,40,55]. Owczarek et al. (2022) studies evaluated the phenolic composition, antioxidant and cytotoxic activity of *A. melanocarpa* leaf extracts, showing selective cytotoxicity toward cancer cell lines associated with significant ROS modulation [62]. This provides evidence that not only fruit extracts but also leaf-derived polyphenols can contribute to oxidative stress regulation in pathological contexts [62].

In addition to their antioxidant role, *Aronia melanocarpa* polyphenols exert potent anti-inflammatory effects [63]. They suppress the expression of key pro-inflammatory mediators such as TNF-α, IL-6 and inducible nitric oxide synthase (iNOS) by downregulating signaling pathways like NF-κB and MAPK [64,65]. Ultrasound-assisted extracts from aronia pomace have demonstrated efficacy in reducing inflammation in vitro, supporting their therapeutic relevance in CRC [64]. Also, valorization of aronia pomace as a by-product has been explored. Lazăr et al. (2020) characterized pomace fractions for functional ingredient development, confirming retained antioxidant capacity and polyphenol potential [66]. Furthermore, encapsulation strategies using hemicellulose matrices have shown promise in enhancing the bioactivity and targeted delivery of polyphenols to inflamed intestinal tissues, thereby improving their potential for managing inflammatory bowel disease and CRC [67].

Interestingly, recent studies suggest that the biological activity of aronia polyphenols may be dose-dependent. While moderate concentrations boost cellular antioxidant capacity, higher doses in cancer cells can paradoxically induce ROS-mediated apoptosis, selectively eliminating malignant cells [61]. Notably, comparative phytochemical profiling reveals that aronia fruits contain significantly higher levels of polyphenols and antioxidant potential than many commonly consumed berries and fruits, highlighting their unique value in functional food and nutraceutical approaches to CRC prevention [37,68].

### 3.2. Modulation of Apoptotic Pathways in CRC Cells

Polyphenols have significant anticancer effects in CRC cells by modulating key apoptotic pathways. One primary mechanism involves the activation of caspase-dependent apoptosis, particularly caspase-3, -8 and -9, which leads to programmed cell death in tumor cells [61,69]. Coinciding, polyphenols influence the balance between pro-apoptotic (e.g., Bax, Bad) and anti-apoptotic proteins (e.g., Bcl-2, Bcl-xL), often tipping the scale toward cell death by promoting mitochondrial membrane permeabilization and cytochrome c release [55].

Specifically, aronia polyphenols—especially anthocyanins and proanthocyanidins—have been shown to downregulate Bcl-2 and upregulate Bax in CRC cells, thereby sensitizing them to apoptosis [40]. This modulation is often mediated via upstream signaling pathways, including the inhibition of PI3K/Akt and NF-κB, which are commonly upregulated in colorectal tumors and linked to cell survival and chemoresistance [61,69,70]. Moreover, some studies report that high concentrations of polyphenols can induce ROS-mediated apoptosis, selectively damaging cancer cells without affecting normal colonocytes, suggesting dual antioxidant/pro-oxidant behavior depending on context and dosage [55,61].

### 3.3. Inhibition of Cancer Cell Proliferation and Metastasis

Polyphenols from natural dietary sources demonstrate strong antiproliferative and antimetastatic effects against CRC cells by modulating key molecular signaling pathways involved in tumor progression (Table 2).

Studies on anthocyanin-rich extracts from *Aronia melanocarpa* fruit have shown suppression of CRC cell growth via downregulation of Cyclin D1, activation of cell cycle inhibitors p21 and p27 and inhibition of PI3K/Akt and MAPK/ERK signaling cascades, which are critical for tumor cell survival and proliferation [55,61]. Additionally, their fruit polyphenols downregulate NF-κB and STAT3, two key regulators of inflammatory and proliferative responses in colorectal carcinogenesis [61].

Crucially, aronia extracts also target mechanisms involved in tumor metastasis. The suppression of matrix metalloproteinases MMP-2 and MMP-9, enzymes essential for extracellular matrix degradation and cancer cell invasion, has been observed following treatment with aronia polyphenols [40]. These compounds also interfere with epithelial–mesenchymal transition (EMT) by increasing the expression of E-cadherin and suppressing mesenchymal markers such as Snail and ZEB1, thereby limiting cancer cell motility and invasiveness [72].

Beyond fruit-derived compounds, recent research has highlighted the anti-metastatic activity of aronia leaf extracts, which similarly reduce CRC cell migration and invasion. This activity has been linked to reduced MMP activity and modulation of EMT-related genes, providing additional evidence for the therapeutic potential of different aronia plant parts [24].

## 4. Role of Polysaccharides in CRC Prevention

### 4.1. Influence on Gut Microbiota and Its Role in CRC Prevention

Recent advances have reinforced the central role of dietary polysaccharides in preventing CRC through modulation of the gut microbiota. Carbohydrates complex serve fermentable substrates for beneficial bacteria such as *Bifidobacterium*, *Lactobacillus* and *Roseburia*, promoting the production of SCFAs, especially butyrate [73,74]. Butyrate, in turn, exerts anti-inflammatory and anti-proliferative effects in colonic epithelial cells, enhancing epithelial barrier integrity, modulating immune responses and inducing apoptosis in dysplastic or cancerous cells [14,73]. A review in the British Journal of Nutrition further emphasizes butyrate’s role in CRC prevention by inhibiting histone deacetylases, reducing pro-inflammatory signaling and downregulating genes involved in tumorigenesis [75]. Moreover, recent findings by Liu et al. (2024) suggest that natural polysaccharides can reshape the gut microbial landscape in favor of anti-tumor immunity, reduce the abundance of pathogenic species and support the efficacy of immunotherapy. These multifaceted microbial and immunomodulatory effects underscore the therapeutic relevance of polysaccharides as functional dietary components in CRC prevention strategies [76].

### 4.2. Anti-Inflammatory and Antioxidant Properties of Polysaccharides

Dietary polysaccharides derived from natural plant sources, often coexisting with polyphenolic compounds such as kaempferol, epicatechin, resveratrol and curcumin, exhibit potent anti-inflammatory and antioxidant properties, making them promising candidates for the prevention and management of chronic intestinal inflammation—a recognized risk factor in the pathogenesis of CRC. These polysaccharide-polyphenol complexes synergistically modulate inflammatory signaling by inhibiting the activation of NF-κB, a key transcription factor involved in the expression of pro-inflammatory cytokines including IL-6 and TNF-α [77,78].

Simultaneously, polysaccharides contribute to oxidative stress reduction by scavenging ROS, stabilizing redox homeostasis and enhancing the activity of endogenous antioxidant enzymes such as SOD and GPx. These mechanisms not only prevent oxidative DNA damage but also support the maintenance of mucosal integrity and attenuate inflammation-induced cytotoxicity in colonic epithelial cells [79]. Findings from in vivo studies corroborate these effects, where supplementation with plant-derived polysaccharides has been shown to reduce inflammatory cell infiltration, preserve epithelial barrier function, and restore intestinal homeostasis [80]. These data underscore the chemopreventive potential of polysaccharide-rich plant extracts, particularly those enriched with complementary phytochemicals, in reducing the burden of CRC through dual modulation of oxidative and inflammatory pathways.

### 4.3. Potential to Inhibit CRC-Related Pathways

Beyond their well-documented anti-inflammatory and antioxidant actions, dietary polysaccharides derived from plants exhibit a multifaceted potential to inhibit molecular pathways directly involved in CRC development and progression. Emerging evidence from recent reviews and experimental studies demonstrates that these polysaccharides can suppress CRC cell proliferation by modulating several oncogenic signaling cascades critical for tumor growth and survival. Notably, polysaccharides have been shown to downregulate the PI3K/Akt pathway, which is often hyperactivated in CRC and contributes to enhanced cell proliferation, survival and chemoresistance [81]. Additionally, they interfere with the IL-6/STAT3 axis, a key driver of chronic inflammation-induced tumorigenesis, thereby reducing pro-survival and pro-inflammatory gene expression that promotes tumor progression.

Polysaccharides also modulate mTOR signaling, a central regulator of cellular metabolism and growth, thus impairing cancer cell anabolism and proliferation. The inhibition of TLR4/JNK pathways further contributes to their anti-tumor effects by suppressing inflammatory responses and inducing apoptosis in cancer cells. These compounds can promote programmed cell death through both apoptosis and autophagy, enhancing the elimination of malignant cells [81]. Importantly, polysaccharides influence non-coding RNAs, including microRNAs and long non-coding RNAs, which regulate intrinsic cell death mechanisms and tumor suppressor gene expression, highlighting a complex layer of epigenetic regulation in CRC chemoprevention.

Moreover, the ability of polysaccharides to inhibit metastatic processes has been linked to the disruption of Smad2/3 and IRS1 signaling, pathways implicated in EMT and cellular migration. By impeding these pathways, polysaccharides reduce tumor invasiveness and dissemination, which are critical steps in CRC progression and patient prognosis [81].

## 5. Polyphenol–Polysaccharide Complexes from *Aronia melanocarpa*: Synergistic Effects

### 5.1. Enhancement of Bioavailability and Stability of Active Compounds

Anthocyanins derived from *Aronia melanocarpa* exhibit significant therapeutic potential; however, their poor stability and low bioavailability under physiological and environmental conditions limit clinical application. Recent strategies based on polysaccharide complexation have demonstrated promising results in enhancing anthocyanin stability. For instance, the use of carboxymethyl cellulose (CMC), pectin and xanthan gum (XG) enabled the formation of stable anthocyanin-polysaccharide complexes through hydrogen bonding, cation-π interactions and electrostatic forces. These complexes markedly improved resistance to heat, light, oxidation, and metal ions, while significantly enhancing antioxidant activity-especially with XG, which also improved bile salt-binding and tyrosinase inhibition [82].

Further advancements include encapsulation techniques using maltodextrin combined with polysaccharides such as CMC, XG, or gum arabic. These formulations achieved encapsulation efficiencies of 94–98%, with anthocyanin retention reaching 88–91% after 100 days of storage at 25 °C, compared to only 47% in non-encapsulated samples. Notably, cyanidin-3-galactoside and cyanidin-arabinosides demonstrated higher stability than their glucoside and xyloside counterparts [83].

A particularly promising approach involves amylopectin nanoparticle delivery systems, which bind anthocyanins more than 80% efficiency. This delivery system increased antioxidant retention, protected anthocyanins from digestive degradation and enhanced intestinal absorption [84]. A subsequent pharmacokinetic study confirmed that amylopectin encapsulation improved oral bioavailability by 440–593%, prolonged plasma retention time and reduced excretion of unmetabolized anthocyanins, demonstrating effective sustained release [85]. Collectively, these strategies represent advanced delivery systems that significantly improve the functional performance of *Aronia melanocarpa* anthocyanins in food, pharmaceutical and nutraceutical formulations.

### 5.2. Combined Action on CRC Prevention and Synergistic Effects

The integration of *Aronia melanocarpa*-derived polyphenols and polysaccharides reveals compelling synergistic mechanisms in CRC prevention. Specifically, Li et al. (2024) demonstrated that the combination of Aronia Berry Extract (ABE) with oligomeric proanthocyanidins (OPCs) significantly reduced CRC cell viability and colony formation, while amplifying apoptosis in vitro and in 3D organoid models. This effect was mechanistically traced to downregulation of lamin B1 (LMNB1) and subsequent suppression of AKT phosphorylation, emphasizing multi-targeted pathway modulation more effective than with either agent alone [86].

Complementing this, Wei et al. (2020) showed that anthocyanins from aronia induce apoptosis in Caco-2 cells by inhibiting the Wnt/β-catenin pathway—specifically, reducing cytoplasmic β-catenin levels, arresting the cell cycle and triggering apoptotic processes [87].

Although anthocyanin–polysaccharide complexes from aronia have not yet been directly tested in CRC models, Wang et al. (2024) provided strong evidence that such complexes substantially enhance anthocyanin stability, antioxidant activity and resistance to degradation—all essential precondition for effective in vivo action. This improved stability likely prolongs anthocyanin exposure to colon cells, thereby strengthening inhibition of oncogenic pathways like Wnt/β-catenin [55].

Altogether, these findings indicate a synergistic interplay: polyphenol-polyphenol combinations (e.g., ABE + OPCs) efficiently suppress oncogenic signaling (LMNB1-AKT), while polysaccharide stabilization enhances persistence and bioactivity of anthocyanins in the colon, permitting more effective engagement of apoptotic and anti-proliferative mechanisms [86,88].

**Figure 1:**
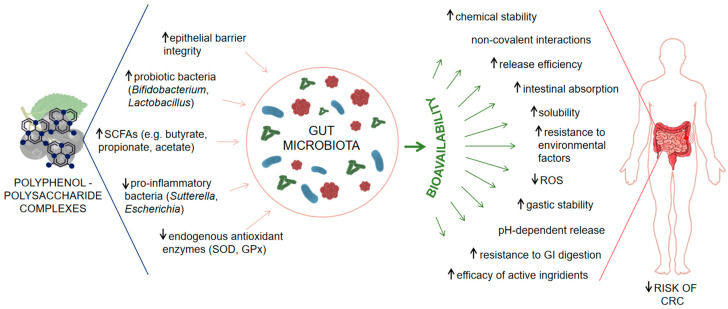
Schematic representation of the modulatory effects of polyphenol–polysaccharide complexes from *Aronia melanocarpa* on the gut microbiota, the bioavailability of polyphenols, and the associated risk of CRC. Polyphenol–polysaccharide complexes modulate the gut microbiota by enhancing epithelial barrier integrity, increasing the abundance of probiotic bacteria (*Bifidobacterium*, *Lactobacillus*) and levels of short-chain fatty acids (SCFAs: butyrate, propionate, acetate), while reducing pro-inflammatory taxa (*Sutterella*, *Escherichia*) and stimulating endogenous antioxidant enzymes such as superoxide dismutase (SOD) and glutathione peroxidase (GPx). These microbiota-mediated effects support intestinal homeostasis and lower inflammation-associated carcinogenic risk. In parallel, complexation with polysaccharides improves polyphenol bioavailability by increasing chemical stability, solubility, pH-dependent and controlled release, resistance to gastrointestinal digestion and environmental stressors, intestinal absorption, and the efficacy of active ingredients, while decreasing ROS levels. The combined microbiota-dependent and bioavailability-enhancing mechanisms contribute to a reduced risk of colorectal cancer.

Figure 1 summarizes supporting evidence explaining how polyphenol–polysaccharide complexes from *Aronia melanocarpa* enhance polyphenol bioavailability and modulate the gut microbiota in the prevention of CRC.

## 6. In Vitro and In Vivo Studies on *Aronia melanocarpa* in CRC Chemoprevention

### 6.1. In Vitro Models: Impact on CRC Cell Lines and Tumor Markers

Cell culture studies have demonstrated that extracts from black chokeberry, rich in polyphenols such as anthocyanins, flavanols and phenolic acids, significantly suppress the proliferation of colorectal cancer cell lines such as Caco-2, HT-29 and SW-480 by inducing apoptosis and causing cell cycle arrest. Bermúdez-Soto et al. (2007) reported that prolonged exposure of Caco-2 cells to chokeberry juice led to the upregulation of the tumor suppressor protein, contributing to reduced cancer cell growth [89]. Likewise, Wei et al. (2020) demonstrated that anthocyanins from *Aronia melanocarpa* suppress the Wnt/β-catenin signaling pathway, resulting in decreased cytoplasmic β-catenin levels and downregulation of pro-proliferative target genes, thereby enhancing cancer cell apoptosis [87].

Further research by Cvetanović et al. (2018) revealed that among different plant parts, leaf extracts showed the strongest anti-metastatic properties by inhibiting cancer cell migration and invasion through downregulation of matrix metalloproteinases MMP-2 and MMP-9, which are essential for extracellular matrix degradation [90]. Similarly, Gill et al. (2021) compared various chokeberry species and confirmed that black chokeberry extract had the highest antiproliferative activity against HT-29 cells, correlating with its high total phenolic content and antioxidant potential [18]. These findings underscore the importance of specific polyphenolic constituents (especially chlorogenic and caffeic acids) in mediating aronia’s anticancer properties [20].

Complementing these earlier studies, Kulling and Rawel (2008) showed that flavanol-rich chokeberry extracts induce cell cycle arrest and apoptosis in CRC cells, while also modulating the expression of genes associated with cellular survival and oxidative stress response [28].

Recent findings by Owczarek et al. (2022) further support these conclusions. In their study, two types of aronia leaf extracts—crude (ACE) and purified (APE)—were tested on CRC cell lines SW-480 and HT-29. The APE extract demonstrated significantly stronger cytotoxic effects in a dose and time-dependent manner, with an IC_50_ of approximately 194 µg/mL for SW-480 cells after 48 h of exposure. Notably, these effects were selective, showing minimal toxicity toward normal colon epithelial cells (CCD 841 CoN), suggesting potential therapeutic relevance. The enhanced activity of APE was attributed to its enriched content of phenolic acids and antioxidant compounds, which likely play a central role in modulating tumor cell viability, inflammation and oxidative stress [62].

In 2024, Stanca and his colleagues provided additional evidence for the anticancer potential of chokeberry by demonstrating that polyphenol and anthocyanin-rich pomace extracts from *Aronia melanocarpa* significantly reduced the viability of CRC cells (C2BBe1) in vitro. Their study revealed that chokeberry extracts exert cytotoxic effects in a dose-dependent manner, modulate key signaling pathways such as Akt and Erk1/2, and downregulate the expression of pro-inflammatory cytokines and matrix metalloproteinases (MMP-1, MMP-2, MMP-3), indicating that their action involves both antiproliferative and anti-inflammatory mechanisms [91].

### 6.2. Animal Model Studies

In vivo studies provide additional support for earlier in vitro findings. Valcheva-Kuzmanova and colleagues provide compelling evidence of the broad-spectrum health-promoting properties of Black chokeberry, particularly in the context of gastrointestinal disorders and metabolic dysfunctions. Investigations demonstrated potential of aronia juice against indomethacin-induced gastric mucosal damage, primarily through suppression of oxidative stress and upregulation of endogenous antioxidant enzymes such as SOD and catalase [92,93,94]. More recent studies using a 2,4,6-Trinitrobenzenesulfonic acid (TNBS)-induced colitis model in rats revealed that aronia juice ameliorates the symptoms of IBD by reducing inflammatory cytokine expression, improving histopathological features of the colon and restoring redox homeostasis [94].

More recent research by Zhao et al. (2021) and Zhu et al. (2022) revealed that aronia-derived polysaccharides positively modulate the gut microbiota by promoting the growth of *Bacteroides* species and increasing SCFAs production. These SCFAs enhance intestinal barrier integrity and regulate inflammatory pathways, notably through AMPK/SIRT1/NF-κB signaling, which are crucial in CRC pathogenesis [41,59]. Similarly, Kaczmarczyk et al. (2025) reviewed animal and human studies confirming that *A. melanocarpa* supplementation reduces oxidative stress and inflammation while improving gut microbiota composition [24].

Additional in vivo models of colitis induced by dextran sodium sulfate or TNBS have demonstrated that aronia extracts reduce pro-inflammatory cytokines (e.g., TNF-α, IL-1β), oxidative stress markers (MDA) and restore endogenous antioxidant enzymes such as catalase and GPx [95,96]. Moreover, these treatments enhanced the expression of tight junction proteins (ZO-1, occludin, claudin-1), thereby improving intestinal barrier function and elevating SCFAs levels, further supporting gut homeostasis [97].

T-cell transfer-induced colitis models provided additional mechanistic insights, showing that dietary supplementation with 4.5% aronia powder attenuated colonic inflammation by reducing TNF-α and IFN-γ levels, lowering MDA and preserving antioxidant defenses (GPx, reduced glutathione, Nfe2l2) in intestinal tissues and associated lymph nodes [98]. Notably, aronia fruit juice demonstrated superior efficacy to sulfasalazine in TNBS-induced colitis rats by significantly ameliorating clinical symptoms and decreasing thiobarbituric acid reactive substances, confirming its potent antioxidant and anti-inflammatory properties [94].

Complementary in vitro experiments with anthocyanin-rich aronia extracts further support these findings by demonstrating suppression of inflammatory mediators IL-1β, TNF-α and MDA in Lipopolysaccharide-stimulated macrophages without cytotoxicity [40]. Collectively, these animal model studies elucidate multiple molecular mechanisms through which aronia exerts therapeutic effects, including modulation of inflammatory signaling (e.g., NF-κB pathway), reduction in oxidative damage, enhancement of gut barrier integrity and beneficial alteration of microbiota composition.

### 6.3. Clinical Trials on Aronia melanocarpa in Cancer Prevention

Clinical and preclinical studies based on *Aronia melanocarpa* extracts have explored its health benefits across various populations, including healthy individuals, athletes, and those with metabolic disorders or cardiovascular conditions. Early clinical evidence demonstrated significant anti-inflammatory effects of aronia supplementation in high-risk populations. In a 6-week placebo-controlled trial, Naruszewicz et al. (2007) found that daily intake of 255 mg of aronia extract significantly reduced high-sensitivity C-reactive protein (hs-CRP) and IL-6 in post-myocardial infarction patients [99]. Similarly, Broncel et al. (2010) reported that 8 weeks of supplementation with 300 mg/day of extract in 25 patients with metabolic syndrome did not significantly alter CRP levels but improved the activity of endogenous antioxidant enzymes such as SOD and GPx, while CAT activity decreased, highlighting enzyme-specific responses [100].

In healthy populations, the effects of aronia are more modest but still evident. A 12-week single-arm study by Kardum et al. (2014) showed increased SOD and GPx activity in 29 healthy women consuming 100 mL of chokeberry juice daily [101]. In hypertensive individuals, a 4-week intervention with 200 mL/day of aronia juice led to a significant reduction in blood pressure and improvements in lipid parameters [102]. Findings in diabetic populations have been more variable. Milutinović et al. (2019) reported that a 12-week intervention with 150 mL/day of juice in type 2 diabetes patients led to improvements in LDL cholesterol, HbA1c, and select hematological parameters, suggesting potential benefits for metabolic and cardiovascular health [103].

More recent trials have strengthened the evidence for aronia’s bioactivity, particularly in metabolically compromised individuals. Tasić et al. (2021) demonstrated that standardized extract for four weeks in 143 participants with metabolic syndrome significantly improved blood pressure, glucose regulation, and lipid profile key metabolic disturbances associated with increased CRC risk [104].

Gancheva et al. (2021), in a 12-week parallel trial in overweight adults, found reduced CRP and increased SOD activity, indicating a decrease in systemic inflammation and oxidative stress. Catalase activity remained largely unchanged, suggesting that aronia’s antioxidant effects may be selective for specific enzymatic pathways [105]. In contrast, Milosavljević et al. (2021) investigated the effects of one-month supplementation with 30 mL/day of a standardized *Aronia melanocarpa* extract in 30 anemic hemodialysis patients. They observed a significant increase in CAT and reduced glutathione levels, along with a decrease in SOD anion, but systemic inflammation was not significantly improved [106].

In physically active individuals, who often experience exercise-induced oxidative and inflammatory stress, aronia also shows potential. Two studies by Stankiewicz et al. (2021, 2023) in male athletes investigated the effects of 7- and 12-week supplementation with either chokeberry juice (200 mL/day) or freeze-dried extract (6 g/day). In the 2023 study, supplementation led to reductions in the pro-inflammatory cytokine IL-6 and increases in the anti-inflammatory cytokine IL-10. However, this effect was not observed in the earlier 2021 trial [107,108].

Chung et al. (2023), in an 8-week trial in healthy individuals following exercise, found increased GPx activity without significant changes in IL-6 or catalase, suggesting antioxidant benefits even without overt inflammation reduction [109]. In populations with low baseline inflammation, results have been mixed. A 90-day randomized crossover trial by Sangild et al. (2023) in hypercholesterolemic adults receiving 150 mg/day of an anthocyanins standardized *Aronia* spp. extract reported a significant increase in glutathione levels and improvement in cytoprotective targets in specific subgroups of men over 40 years of age [110].

Among the most recent findings, Chamberlin et al. (2024) conducted a 30-day randomized, placebo-controlled trial in 14 healthy adults, showing that daily intake of 100 mL of polyphenol-rich aronia juice significantly reduced postprandial glucose (β = −3.03, *p* < 0.01), prevented increases in fasting total cholesterol (β = −0.50, *p* = 0.03), and modulated both serum and gut metabolites involved in central carbon and lipid metabolism. Importantly, aronia consumption was associated with decreased levels of pro-inflammatory metabolites, underscoring its systemic anti-inflammatory effects [22].

To consolidate the growing body of evidence, Sarıkaya et al. (2025) conducted a systematic review of 18 randomized controlled trials published between 2022 and 2023. The review evaluated interventions using aronia juice, extract and also over-dried powder. The results confirm consistent reductions in inflammatory markers such as TNF-α, IL-6, and CRP, as well as significant enhancements in antioxidant enzyme activity (SOD, CAT, GPx) across diverse populations [23]. These findings are consistent with broader clinical evidence on the role of polyphenol-rich foods as summarized in a recent systematic review by López-Gómez & Uranga (2024). Dietary polyphenols, including those found in *Aronia melanocarpa*, have shown promising results in the prevention and adjunctive treatment of CRC, primarily through anti-inflammatory pathways, modulation of gut microbiota, and maintenance of epithelial barrier integrity [42]. Furthermore, a meta-analysis by Xu et al. (2021) demonstrates that dietary polyphenol supplementation significantly affects iron metabolism and erythropoiesis across diverse populations, suggesting that polyphenols can modulate physiological parameters relevant to cancer risk in a context-dependent manner [111].

## 7. Study Limitations and Future Perspectives

*Aronia melanocarpa* is a promising functional food ingredient due to its exceptionally high content of polyphenols, including anthocyanins, proanthocyanidins, and phenolic acids. However, its practical application faces some challenges. One of the main considerations is the relatively low bioavailability of these compounds, which may limit their biological efficacy in humans. Many polyphenols are only partially absorbed in the gastrointestinal tract and subsequently metabolized or degraded during processing and storage. Encapsulation strategies, such as binding anthocyanins to amylopectin nanoparticles, enhance systemic bioavailability in vivo [82,84,85].

Another important factor is the variability of bioactive composition in the raw material—the polyphenol profile can differ depending on the aronia cultivar, cultivation conditions, fruit maturity, and extraction methods, making it difficult to standardize preparations and compare clinical study results [30,37].

Technological and nutritional aspects can also affect their practical use: Aronia extracts are naturally astringent and bitter, which may influence consumer acceptance in food products. Strategies such as microencapsulation or freeze-drying can improve the stability of phenolic compounds and their bioavailability, though they may add complexity to production and formulation [67,112].

Despite strong mechanistic support, meta-analyses of randomized human trials suggest that clinical evidence for some of aronia’s health benefits remains limited or variable. For example, effects on cardiometabolic markers are promising but not always consistent, with many studies featuring small sample sizes, short intervention periods, and differences in dosage. Regulatory aspects also need consideration, as the classification of Aronia extracts—whether as a functional food, dietary supplement, or medicinal ingredient—can influence requirements for clinical testing, labeling, and health claims [23,42].

In terms of safety, *Aronia melanocarpa* is generally well tolerated. Nevertheless, some aspects deserve attention, particularly regarding long-term or high-dose intake. The polyphenols and tannins naturally present in chokeberry may modestly reduce non-heme iron absorption, an effect supported by studies and meta-analyses of polyphenol supplementation [113,114]. While this is unlikely to be clinically significant for most healthy adults, it may be relevant for populations at risk of iron deficiency.

Additionally, the literature indicates that some polyphenols exhibit prooxidant activity at high concentrations, resulting in increased production of reactive oxygen species and induction of apoptosis in cancer cells. Mechanistically, this effect may result from autoxidation of phenolic compounds, redox cycling involving transition metals, and inhibition of cellular antioxidant systems [115]. It is essential to note that most reports of prooxidant activity originate from in vitro studies conducted at concentrations significantly higher than those typically achievable after oral administration (i.e., studies ranging from tens to hundreds of µM or even mM), whereas plasma concentrations following dietary polyphenol consumption are generally in the µM range or lower [116,117]. Furthermore, in vivo factors such as hepatic metabolism, plasma protein binding, and transformation by gut microbiota significantly influence the biological activity and availability of free polyphenols. This phenomenon can be considered in the context of hormesis: low doses of polyphenols often exhibit cytoprotective and antioxidant effects, whereas higher doses can induce oxidative stress and apoptosis [62]. This has two practical implications. First, translational research should carefully assess the transfer of results from in vitro models to in vivo and then to humans; second, prooxidant effects at high doses may have therapeutic applications (e.g., targeted destruction of cancer cells) but also carry the risk of toxicity to normal cells and potential drug interactions (e.g., during chemotherapy). Therefore, detailed pharmacokinetic and toxicological studies are necessary to establish safe exposure ranges and potential dose or concentration thresholds for different preparations and clinical settings [113,118].

Finally, mild gastrointestinal effects, such as temporary astringency or discomfort, have occasionally been reported in studies using higher doses of chokeberry products [119]. Since most clinical trials have been relatively short (<12 weeks), future studies would benefit from monitoring long-term safety and potential impacts on micronutrient status.

## 8. Conclusions

*Aronia melanocarpa* (Michx.) Elliot exhibits multifaceted health-promoting properties, particularly in the context of inflammation-associated disorders such as CRC. Its berries contain high levels of anthocyanins (e.g., cyanidin-3-galactoside, cyanidin-3-arabinoside), phenolic acids (notably chlorogenic acid) and flavonoids such as quercetin and epicatechin, all of which exhibit strong antioxidant, anti-inflammatory and antiproliferative effects through mechanisms such as NF-κB inhibition, induction of apoptosis and modulation of gene expression. In addition, aronia polysaccharides (e.g., pectins, arabinogalactans) support gut microbiota by promoting beneficial taxa such as *Bacteroides*, increasing SCFAs production and strengthening intestinal barrier integrity. Together, these synergistic actions help reduce oxidative stress, inflammation and epithelial permeability—key features of CRC pathogenesis.

Polyphenols and polysaccharides thus complement one another: polyphenols reduce oxidative and inflammatory processes, while polysaccharides improve microbiota composition, thereby enhancing polyphenol metabolism and bioavailability, and resulting in a mutually reinforcing anti-inflammatory and anti-tumorigenic effect relevant to CRC prevention.

Beyond fresh fruits, *Aronia* by-products such as pomace (pressing residue) offer an underutilized but potent source of polyphenols and fibers. Pomace retains a significant fraction of anthocyanins and proanthocyanidins (up to 40% of total antioxidant activity) and exhibits superior radical scavenging ability compared to many other fruit-based matrices. Seasonal harvesting and juice processing generate large amounts of pomace, making it an economically attractive raw material for functional foods, nutraceuticals and pharmaceutical formulations. Leaf extracts, although less studied, have also shown antioxidant and antiproliferative activity, offering an additional seasonal fraction with potential biomedical value. These characteristics suggest clear practical applications: pomace and leaves can be incorporated into fiber-enriched formulations, polyphenol-rich dietary supplements, or functional food matrices designed to modulate inflammation and support gut health, thereby offering translational potential for CRC chemoprevention.

However, data specifically evaluating aronia pomace in CRC prevention are still lacking, highlighting a critical research gap. Bioavailability also presents a challenge, as polyphenols are susceptible to degradation during processing and digestion. Recent studies suggest that encapsulation technologies (e.g., zein nanoparticles, spray-drying) may help preserve their stability and activity throughout the gastrointestinal tract.

*Aronia melanocarpa* is generally recognized as safe and well tolerated, although some considerations regarding long-term or high-dose intake should be noted. Polyphenols and tannins naturally occurring in chokeberry may modestly reduce non-heme iron absorption, which could be relevant for populations at risk of iron deficiency, though clinically significant effects in healthy individuals are unlikely. Occasional mild gastrointestinal discomfort has been reported in studies using higher doses of chokeberry products, and potential interactions with drug-metabolizing enzymes remain theoretical, as no serious adverse events have been documented. Including routine safety monitoring in future long-term intervention trials would strengthen the evidence base for aronia supplementation.

In summary, the combination of potent phytochemicals, microbiota-modulating polysaccharides, and a growing body of scientific evidence indicates that *Aronia melanocarpa* as a promising dietary component for the prevention and potentially as an adjunctive treatment of CRC and other inflammation-related malignancies. The valorization of pomace and leaves enhances year-round applicability and supports sustainable production of bioactive-rich materials for health-oriented purpose applications. While its safety profile appears favorable, further research is warranted to confirm long-term tolerability, optimize polyphenol bioavailability, and conduct more rigorous and extended human trials to translate preclinical results into measurable health benefits.

## Figures and Tables

**Table 1:** Quantitative Composition of Polyphenolic and Bioactive Compounds of *Aronia melanocarpa*: Whole fruit, juice, leaves and pomace. All values are approximated per 100 g dry weight or equivalent dry mass for consistency and derived from peer-reviewed studies. DP, Degree polymerization; DW, dry weight; GAE, Gallic Acid Equivalents; QE, quercetin equivalent; CGE, Cyanidin-3-glucoside equivalents.

Compound Class	Whole Fruit	Juice	Leaves	Pomace	Ref.
	mg/g * GAE; ** QE, *** CGE				
Total Polyphenols	78.5 DW ^a^ 28 * DW ^b^ 7–25.6 * FW ^c^ 10 * FW ^d^	37.3 DW ^a^ 9.15 ^e^ 3–6.6 * ^d,f^	61 * DW ^b,d^	105.8 DW ^a^ 23 * DW ^b^ 10.2 * FW ^d^ 15–53 * DW ^d,f^	[25,27,28,29,30,31,32,33]
Total Flavonoid	5.2 ** DW ^b^	-	8.5 ** DW ^b^	190 ** DW ^b^	[28,30]
Anthocyanins (e.g., Cyanidin- -3-galactoside, -3-arabinoside, -3-glucoside)	19.6 DW ^a^ 2.8 DW ^g^	11.7 DW ^a^ 0.15–1.23 *** ^f^	0.52 DW ^h^ 0.02 DW ^g^	18.4 DW ^a^ 1.4–24.7 *** DW ^f^	[26,27,28,29,34,35,36]
Flavonols (e.g., quercetin-3-rutinoside, quercetin-3-glucoside)	0.12 DW ^g^ 1.1–1.4 DW ^g^ 1 DW ^a^ 5.2 ** DW ^b^	1.55 DW ^a^	1.8–7.9 DW ^d^ 2.9 DW ^g^ 8.5 ** DW ^b^	1.7 DW ^a^	[16,27,29,30,36]
Phenolic Acids (e.g., chlorogenic acid, neochlorogenic acid)	5.9 DW ^a^ 2.9–4.9 DM ^g^	8.10 DW ^a^	13.1 DW ^h^ 7.8–12 DW ^g^	3.7 DW ^a^	[27,35,36]
Proanthocy-anidins (e.g., epicatechin-type B-type dimers and polymers)	51.8 DW ^a^ DP = 23	15.8 DW ^a^ DP = 23	Data limited	82 DW ^a^ DP = 34	[27]
Dietary Fiber (e.g., pectin, hemicellulose)	56 FW ^d^	Trace ^d^	Data limited	189–234 FW ^d^ 578 DM ^i^ 958 DM ^d^	[16,28,34,37]
Organic Acids (e.g., citric, malic, quinic acids)	16 FW ^d^ 8.2–17 DM ^d^	1.3–1.9 ^d^ 12–22 DM ^d^	Data limited	0.5–0.6 ^d^	[28,37]
Vitamin C	0.013–0.27 FW ^d^	0.2 FW ^d^	Data limited	Data limited	[28,37]
Minerals (e.g., Na, K, Mg, Ca, Fe, Zn)	0.08 DW ^b^ 4–6 DW ^d^ 2.7 FW ^d^	2.2–5.5 ^d^ 5 DW ^d^	0.3 DW ^b^ 8.8 DW ^d^	0.12 DW ^b^ 14–39 DW ^d^	[28,30]

Legend: Values are reported as in the original studies. Data may be given on a fresh weight (FW) or dry weight (DW) basis, depending on the source, because moisture content was not consistently available for conversion. The broad ranges observed for some compounds result from differences in ripening stages, environmental conditions, extraction methods, and analytical procedures. Consequently, inter-study comparisons should be made with caution. Indexes “^a–i^” refer describe sample preparation methods used prior to analysis: ^a^ freezing, thawing, crushing; extraction: methanol/water/acetic acid (70:28:2), homogenization, centrifugation; analysis: HPLC-DAD (anthocyanins, phenolics), Folin–Ciocalteu. ^b^ drying, grinding; extraction: 80% methanol; analysis: HPLC-DAD, Folin–Ciocalteu, AlCl_3_ assay (flavonoids); includes in vitro bioaccessibility testing. ^c^ Extraction with 50–80% methanol/ethanol, acidified solvents; analysis by Folin–Ciocalteu and HPLC-DAD. ^d^ lyophilization; hydroalcoholic extraction; analysis: Folin–Ciocalteu, HPLC, spectrophotometry; ^e^ homogenization of fresh fruits; extraction:methanol:acetone:water (70:20:10); analysis: spectrophotometry, HPLC; ^f^ drying at 40–45 °C, grinding (fruits & pomace); juice analyzed directly; extraction: 80% methanol, sonication/mixing, filtration; pomace powders and capsules; analysis: Folin–Ciocalteu, HPLC-DAD, pH differential method; ^g^ harvesting July vs. September; lyophilization, grinding to powder; extraction: 80% methanol + 0.1% formic acid, sonication; analysis: HPLC-PDA/ESI-MS; ^h^ drying, grinding; extraction:methanol:acetone:water (70:20:10), homogenization, centrifugation; analysis: HPLC-DAD for chlorogenic acids, anthocyanins, sorbitol; ^i^ 50 drying, grinding; extraction: 70% methanol; analysis: AOAC 991.43, 993.19 (dietary fiber) [38,39], Folin–Ciocalteu, HPLC; includes pomace extrusion.

**Table 2:** Molecular mechanisms of action of *Aronia melanocarpa* extracts relevant to colorectal cancer prevention and progression. COX, Cyclooxygenase-2; IL-6, interleukin-6; iNOS, inducible Nitric Oxide Synthase; MMP, matrix metalloproteinase; NF-κB, Nuclear Factor kappa B; p21, protein 21; p27, protein 27; PI3K, PhosphoInositide 3-Kinase; ROS: Reactive Oxygen Species; STAT3, Signal Transducer and Activator of Transcription 3; TNF-α, tumor necrosis factor alpha; VEGF, Vascular Endothelial Growth Factor.

Extract Source	Active Compounds	Molecular Targets/Pathways	Biological Effects	Ref.
Aronia fruits	Anthocyanins: cyanidin-3-galactoside, cyanidin-3-arabinoside, cyanidin-3-glucoside, cyanidin-3-xyloside Proanthocyanidins: epicatechin, catechin dimers and polymers Phenolic acids: chlorogenic acid, neochlorogenic acid, caffeic acid	↓ Cyclin D1 ↑ p21/p27 ↓ PI3K/Akt ↓ Wnt/β-catenin	↓ Proliferation ↑ Cell cycle arrest ↑ Apoptosis	[25,29,40]
	Anthocyanins: cyanidin derivatives (mainly cyanidin-3-galactoside and -3-arabinoside) Flavonols: quercetin, quercetin-3-rutinoside (rutin), kaempferol	↓ NF-κB, ↓ COX-2 ↓ STAT3	↓ Inflammation ↑ Apoptosis sensitivity	[55,71]
Aronia leaves	Polyphenols: chlorogenic acid, neochlorogenic acid, caffeic acid, rutin, isoquercitrin, quercetin, kaempferol Tannins: proanthocyanidins	↓ MMP-2 ↓ MMP-9 ↓ VEGF	↓ Migration and invasion ↓ Metastasis	[62]
	Polyphenols: quercetin, chlorogenic acid, neochlorogenic acid, caffeic acid, ferulic acid, rutin	↓ NF-κB ↓ IL-6 ↓ TNF-α ↓ iNOS	↓ Inflammatory response ↓ Cytokine production	[62,66]
Aronia pomace	Polyphenols: chlorogenic acid, caffeic acid, ferulic acid, quercetin derivatives Fiber-bound proanthocyanidins (condensed tannins) linked with pectin and hemicellulose.	↑ Targeted delivery via hemicellulose encapsulation	Enhanced anti-inflammatory and antioxidant activity	[67]
Aronia polyphenols (general)	Anthocyanins: cyanidin glycosides Flavonoids: quercetin, rutin, kaempferol Phenolic acids: chlorogenic, caffeic Tannins: proanthocyanidins	Dose-dependent ↑ ROS ↑ cancer cell apoptosis	Selective toxicity toward cancer cells, ↑ Mitochondrial stress	[61,68]

Legend: ↑ activation or increase; ↓ inhibition or decrease.

## Data Availability

No new data were created or analyzed in this study. Data sharing is not applicable to this article.

## Abbreviations

CRC, colorectal cancer; COX, DW, dry weight; FW, Fresh Weight; GPx, glutathione peroxidase; IBD, inflammatory bowel diseases; IL-6, interleukin-6; iNOS, inducible Nitric Oxide Synthase; MDA, Malondialdehyde—marker of oxidative stress; MMP, matrix metalloproteinase; NF-κB, Nuclear Factor kappa B; ROS: Reactive Oxygen Species; SCFAs, short-chain fatty acids; TNF-α, tumor necrosis factor alpha; XG, Xanthan Gum

## References

[1] Sung H., Ferlay J., Siegel R.L., Laversanne M., Soerjomataram I., Jemal A., Bray F. (2021). Global cancer statistics 2020: GLOBOCAN estimates of incidence and mortality worldwide for 36 cancers in 185 countries. CA Cancer J. Clin..

[2] Morgan E., Arnold M., Gini A., Lorenzoni V., Cabasag C.J., Laversanne M., Vignat J., Ferlay J., Murphy N., Bray F. (2023). Global burden of colorectal cancer in 2020 and 2040: Incidence and mortality estimates from GLOBOCAN. Gut.

[3] World Cancer Research Fund, American Institute for Cancer Research (2018). Diet, Nutrition, Physical Activity and Colorectal Cancer.

[4] Zheng J., Zhao L., Dong J., Giovannucci E. (2025). Development and validation of Comprehensive Inflammatory Lifestyle Score and association with colorectal cancer risk. Cancer Epidemiol. Biomark. Prev..

[5] Abdalla M., Eberhardson M., Landerholm K., Andersson R.E., Myrelid P. (2025). Impact of inflammatory bowel disease and primary sclerosing cholangitis on colorectal cancer risk: National cohort study. Clin. Gastroenterol. Hepatol..

[6] Iacucci M., Nardone O.M., Ditonno I., Capobianco I., Pugliano C.L., Maeda Y., Majumder S., Zammarchi I., Santacroce G., Ghosh S. (2025). Advancing inflammatory bowel disease-driven colorectal cancer management: Molecular insights and endoscopic breakthroughs towards precision medicine. Clin. Gastroenterol. Hepatol..

[7] Dekker E., Tanis P.J., Vleugels J.L.A., Kasi P.M., Wallace M.B. (2019). Colorectal cancer. Lancet.

[8] Arnold M., Sierra M.S., Laversanne M., Soerjomataram I., Jemal A., Bray F. (2017). Global patterns and trends in colorectal cancer incidence and mortality. Gut.

[9] Katona B.W., Weiss J.M. (2020). Chemoprevention of colorectal cancer. Gastroenterology.

[10] Delgado-Gonzalez P., Garza-Treviño E.N., de la Garza Kalife D.A., Quiroz Reyes A., Hernández-Tobías E.A. (2023). Bioactive compounds of dietary origin and their influence on colorectal cancer as chemoprevention. Life.

[11] Ran Y., Li F., Xu Z., Zeng K., Ming J. (2024). Recent advances in dietary polyphenols (DPs): Antioxidant activities, nutrient interactions, delivery systems, and potential applications. Food Funct..

[12] Wang M., Wang Y., Zhang H. (2025). Dietary polyphenols for tumor therapy: Bioactivities, nano-therapeutic systems and delivery strategies. Food Funct..

[13] Shanmugam M.K., Warrier S., Kumar A.P., Sethi G., Arfuso F. (2015). Potential role of natural compounds as anti-angiogenic agents in cancer. Curr. Vasc. Pharmacol..

[14] Louis P., Hold G.L., Flint H.J. (2014). The gut microbiota, bacterial metabolites and colorectal cancer. Nat. Rev. Microbiol..

[15] Ren Y., Frank T., Meyer G., Lei J., Grebenc J.R., Slaughter R., Gao Y.G., Kinghorn A.D. (2022). Potential benefits of black chokeberry (*Aronia melanocarpa*) fruits and their constituents in improving human health. Molecules.

[16] Jurendić T., Ščetar M. (2021). *Aronia melanocarpa* products and by-products for health and nutrition: A review. Antioxidants.

[17] Go M.Y., Kim J., Jeon C.Y., Shin D.W. (2024). Functional activities and mechanisms of *Aronia melanocarpa* in our health. Curr. Issues Mol. Biol..

[18] Gill N.K., Rios D., Osorio-Camacena E., Mojica B.E., Kaur B., Soderstrom M.A., Gonzalez M., Plaat B., Poblete C., Kaur N. (2021). Anticancer effects of extracts from three different chokeberry species. Nutr. Cancer.

[19] Owczarek K., Caban M., Sosnowska D., Kajszczak D., Lewandowska U. (2024). The Anti-Metastatic Potential of Aronia Leaf Extracts on Colon Cancer Cells. Nutrients.

[20] Sreedharan S., Nair V., Bhargava P., Cisneros-Zevallos L. (2025). Protective Role of Polyphenols from Aronia Berry (*Aronia melanocarpa*) Against LPS-Induced Inflammation in Colon Cells and Macrophages. Nutrients.

[21] Sharma E., Attri D.C., Sati P., Dhyani P., Szopa A., Sharifi-Rad J., Hano C., Calina D., Cho W.C. (2022). Recent updates on anticancer mechanisms of polyphenols. Front. Cell Dev. Biol..

[22] Chamberlin M.L., Peach J.T., Wilson S.M.G., Miller Z.T., Bothner B., Walk S.T., Yeoman C.J., Miles M.P. (2024). Polyphenol-Rich *Aronia melanocarpa* Fruit Beneficially Impact Cholesterol, Glucose, and Serum and Gut Metabolites: A Randomized Clinical Trial. Foods.

[23] Sarıkaya B., Kolay E., Guney-Coskun M., Yiğit-Ziolkowski A., Aktaç Ş. (2025). The effect of black chokeberry (*Aronia melanocarpa*) on human inflammation biomarkers and antioxidant enzymes: A systematic review of randomized controlled trials. Nutr. Rev..

[24] Kaczmarczyk S., Dziewiecka H., Pasek M., Ostapiuk-Karolczuk J., Kasperska A., Skarpańska-Stejnborn A. (2025). Effects of black chokeberry (*Aronia melanocarpa*) supplementation on oxidative stress, inflammation and gut microbiota: A systematic review of human and animal studies. Br. J. Nutr..

[25] Jurikova T., Mlcek J., Skrovankova S., Sumczynski D., Sochor J., Hlavacova I., Snopek L., Orsavova J. (2017). Black chokeberry (*Aronia melanocarpa*) as an important source of bioactive compounds in the prevention of chronic diseases. Molecules.

[26] Kaloudi T., Tsimogiannis D., Oreopoulou V. (2022). *Aronia melanocarpa*: Identification and exploitation of its phenolic components. Molecules.

[27] Oszmiański J., Wojdyło A. (2005). *Aronia melanocarpa* phenolics and their antioxidant activity. Eur. Food Res. Technol..

[28] Kulling S.E., Rawel H.M. (2008). Chokeberry (*Aronia melanocarpa*)—A review on the characteristic components and potential health effects. Planta Med..

[29] Tolić M.T., Jurčević I.L., Krbavčić I.P., Marković K., Vahčić N. (2015). Phenolic content, antioxidant capacity and quality of chokeberry (*Aronia melanocarpa*) products. Food Technol. Biotechnol..

[30] Saracila M., Untea A.E., Oancea A.G., Varzaru I., Vlaicu P.A. (2024). Comparative analysis of black chokeberry (*Aronia melanocarpa* L.) fruit, leaves, and pomace for their phytochemical composition, antioxidant potential, and polyphenol bioaccessibility. Foods.

[31] Jakobek L., Šeruga M., Medvidović-Kosanović M., Novak I. (2007). Antioxidant activity and polyphenols of *Aronia* in comparison to other berry species. Agric. Conspect. Sci..

[32] Rätsep R., Maante-Kuljus M., Karp K., Kaldmäe H., Põldma P., Koort A., Mainla L., Moor U. (2024). Polyphenol composition and antioxidant activity of wine raw materials and pomace from hybrid grapes, aronia, and Japanese quince. Agric. Food Sci..

[33] Zielińska A., Siudem P., Paradowska K., Gralec M., Kaźmierski S., Wawer I. (2020). *Aronia melanocarpa* fruits as a rich dietary source of chlorogenic acids and anthocyanins: ^1^H-NMR, HPLC-DAD, and chemometric studies. Molecules.

[34] Schmid V., Steck J., Mayer-Miebach E., Behsnilian D., Bunzel M., Karbstein H.P., Emin M.A. (2021). Extrusion processing of pure chokeberry (*Aronia melanocarpa*) pomace: Impact on dietary fiber profile and bioactive compounds. Foods.

[35] Zielińska A., Bryk D., Paradowska K., Wawer I. (2020). *Aronia melanocarpa* leaves as a source of chlorogenic acids, anthocyanins, and sorbitol, and their anti-inflammatory activity. Pol. J. Food Nutr. Sci..

[36] Szopa A., Kokotkiewicz A., Kubica P., Banaszczak P., Wojtanowska-Krośniak A., Krosniak M., Marzec-Wróblewska U., Badura A., Zagrodzki P., Bucinski A. (2017). Comparative analysis of different groups of phenolic compounds in fruit and leaf extracts of *Aronia* sp.: *A. melanocarpa*, *A. arbutifolia*, and *A.* × *prunifolia* and their antioxidant activities. Eur. Food Res. Technol..

[37] Sidor A., Gramza-Michałowska A. (2019). Black Chokeberry *Aronia melanocarpa* L.—A Qualitative Composition, Phenolic Profile and Antioxidant Potential. Molecules.

[38] Latimer G.W.J. (2023). AOAC Official Method 991.43. Total, Soluble, and Insoluble Dietary Fiber in Foods: Enzymatic-Gravimetric Method, MES-TRIS Buffer. Official Methods of Analysis of AOAC INTERNATIONAL.

[39] Latimer G.W.J. (2023). AOAC Official Method 993.19. Soluble Dietary Fiber in Food and Food Products: Enzymatic–Gravimetric Method (Phosphate Buffer). Official Methods of Analysis of AOAC INTERNATIONAL.

[40] Banach M., Wiloch M., Zawada K., Cyplik W., Kujawski W. (2020). Evaluation of antioxidant and anti-inflammatory activity of anthocyanin-rich water-soluble Aronia dry extracts. Molecules.

[41] Zhao Y., Liu X., Zheng Y., Liu W., Ding C. (2021). *Aronia melanocarpa* polysaccharide ameliorates inflammation and aging in mice by modulating the AMPK/SIRT1/NF-κB signaling pathway and gut microbiota. Sci. Rep..

[42] López-Gómez L., Uranga J.A. (2024). Polyphenols in the Prevention and Treatment of Colorectal Cancer: A Systematic Review of Clinical Evidence. Nutrients.

[43] Górka K., Terlikowska K.M. *Zastosowanie Polifenoli w Profilaktyce i Leczeniu Nadciśnienia Tętniczego*; Uniwersytet Medyczny w Białymstoku: Białystok, Poland, 2025. https://www.umb.edu.pl/photo/pliki/Dziekanat-WNOZ/monografie/2025/zastosowanie_polifenoli_w_profilaktyce_i_leczeniu_nadcisnienia_tetniczego.pdf.

[44] Chen L., Chen W., Li D., Liu X. (2023). Anthocyanin and proanthocyanidin from *Aronia melanocarpa* (Michx.) Ell.: Purification, fractionation, and enzyme inhibition. Food Sci. Nutr..

[45] Efenberger-Szmechtyk M., Nowak A., Czyżowska A., Kucharska A.Z., Fecka I. (2020). Composition and antibacterial activity of *Aronia melanocarpa* (Michx.) Elliot, *Cornus mas* L. and *Chaenomeles superba* Lindl. leaf extracts. Molecules.

[46] Mohana S., Ganesan M., Agilan B., Karthikeyan R., Srithar G., Beaulah Mary R., Ananthakrishnan D., Velmurugan D., Rajendra Prasad N., Ambudkar S.V. (2016). Screening dietary flavonoids for the reversal of P-glycoprotein-mediated multidrug resistance in cancer. Mol. Biosyst..

[47] Bushmeleva K., Vyshtakalyuk A., Terenzhev D., Belov T., Nikitin E., Zobov V. (2022). Antioxidative and immunomodulating properties of *Aronia melanocarpa* extract rich in anthocyanins. Plants.

[48] Braumüller H., Mauerer B., Andris J., Berlin C., Wieder T., Kesselring R. (2022). The cytokine network in colorectal cancer: Implications for new treatment strategies. Cells.

[49] Ceylan F.D., Günal-Köroğlu D., Saricaoglu B., Ozkan G., Capanoglu E., Calina D., Sharifi-Rad J. (2025). Anticancer potential of hydroxycinnamic acids: Mechanisms, bioavailability, and therapeutic applications. Naunyn-Schmiedeberg’s Arch. Pharmacol..

[50] Baldi S., Tristán Asensi M., Pallecchi M., Sofi F., Bartolucci G., Amedei A. (2023). Interplay between lignans and gut microbiota: Nutritional, functional and methodological aspects. Molecules.

[51] Mompeo O., Spector T.D., Matey Hernandez M., Le Roy C., Istas G., Le Sayec M., Mangino M., Jennings A., Rodriguez-Mateos A., Valdes A.M. (2020). Consumption of stilbenes and flavonoids is linked to reduced risk of obesity independently of fiber intake. Nutrients.

[52] Olas B., Kedzierska M., Wachowicz B., Stochmal A., Oleszek W. (2010). Effects of polyphenol-rich extract from berries of *Aronia melanocarpa* on the markers of oxidative stress and blood platelet activation. Platelets.

[53] Olas B., Wachowicz B., Nowak P., Kędzierska M. (2008). The antiplatelet properties of polyphenol-rich extracts from *Aronia melanocarpa* berries in vitro. Thromb. Res..

[54] Gupta S.C., Patchva S., Aggarwal B.B. (2013). Therapeutic roles of curcumin: Lessons learned from clinical trials. AAPS J..

[55] Wang J., Wang J., Hao J., Jiang M., Zhao C., Fan Z. (2024). Antioxidant activity and structural characterization of anthocyanin–polysaccharide complexes from *Aronia melanocarpa*. Int. J. Mol. Sci..

[56] Dong J., Wang L., Bai Y., Huang X., Chen C., Liu Y. (2024). Study on the physicochemical properties and immune regulatory mechanism of polysaccharide fraction from *Aronia melanocarpa* fruit. Int. J. Biol. Macromol..

[57] Vamanu E., Gatea F., Avram I., Radu G.L., Singh S.K. (2023). Dysbiotic Gut Microbiota Modulation by Aronia Fruits Extract Administration. Life.

[58] Ruiz-Álvarez B.E., Cattero V., Desjardins Y. (2025). Prebiotic-like Effects of Proanthocyanidin-Rich Aronia Extract Supplementation on Gut Microbiota Composition and Function in the Twin-M-SHIME^®^ Model. Pharmaceuticals.

[59] Zhu Y., Wei Y.L., Karras I., Cai P.J., Xiao Y.H., Jia C.L., Qian X.L., Zhu S.Y., Zheng L.J., Hu X. (2022). Modulation of the gut microbiota and lipidomic profiles by black chokeberry (*Aronia melanocarpa* L.) polyphenols via the glycerophospholipid metabolism signaling pathway. Front. Nutr..

[60] Shahidi F., Athiyappan K.D. (2025). Polyphenol-polysaccharide interactions: Molecular mechanisms and potential applications in food systems—A comprehensive review. Food Prod. Process Nutr..

[61] Long J., Guan P., Hu X., Yang L., He L., Lin Q., Luo F., Li J., He X., Du Z. (2021). Natural polyphenols as targeted modulators in colon cancer: Molecular mechanisms and applications. Front. Immunol..

[62] Owczarek K., Sosnowska D., Kajszczak D., Lewandowska U. (2022). Evaluation of phenolic composition, antioxidant and cytotoxic activity of *Aronia melanocarpa* leaf extracts. J. Physiol. Pharmacol..

[63] Zapolska-Downar D., Bryk D., Małecki M., Hajdukiewicz K., Sitkiewicz D. (2012). *Aronia melanocarpa* fruit extract exhibits anti-inflammatory activity in human aortic endothelial cells. Eur. J. Nutr..

[64] Aprodu I., Chitescu C.L., Grigore-Gurgu L., Dumitrașcu L. (2025). Investigation of the antioxidant and antimicrobial properties of ultrasound-assisted extracted phenolics from *Aronia melanocarpa* pomace. Appl. Sci..

[65] Chojnacka K., Lewandowska U. (2021). The influence of polyphenol-rich extracts on the production of pro-inflammatory mediators in macrophages. J. Physiol. Pharmacol..

[66] Lazăr M.-A., Catană M., Catană L., Burnete A.-G., Teodorescu R.I., Asănică A.C., Belc N. (2020). Valorisation of *Aronia melanocarpa* pomace for development of functional ingredients with high nutritional value and antioxidant capacity. Sci. Pap. Ser. B Hortic..

[67] Caban M., Lewandowska U. (2023). Encapsulation of polyphenolic compounds based on hemicelluloses to enhance treatment of inflammatory bowel diseases and colorectal cancer. Molecules.

[68] Teleszko M., Wojdyło A. (2015). Comparison of phenolic compounds and antioxidant potential between selected edible fruits and their leaves. J. Funct. Foods.

[69] Patra S., Pradhan B., Nayak R., Behera C., Das S., Patra S.K., Efferth T., Jena M., Bhutia S.K. (2021). Dietary polyphenols in chemoprevention and synergistic effect in cancer: Clinical evidences and molecular mechanisms. Phytomedicine.

[70] Tosi D., Pérez-Gracia E., Atis S., Vié N., Combès E., Gabanou M., Larbouret C., Jarlier M., Mollevi C., Torro A. (2018). Rational development of synergistic combinations of chemotherapy and molecular targeted agents for colorectal cancer treatment. BMC Cancer.

[71] Sidor A., Gramza-Michałowska A. (2019). Black Chokeberry (*Aronia melanocarpa*) and Its Products as Potential Health-Promoting Factors—An Overview. Trends Food Sci. Technol..

[72] Amawi H., Ashby C.R., Samuel T., Peraman R., Tiwari A.K. (2017). Polyphenolic Nutrients in Cancer Chemoprevention and Metastasis: Role of the Epithelial-to-Mesenchymal (EMT) Pathway. Nutrients.

[73] Fernández J., Redondo-Blanco S., Miguélez E.M., Villar C.J., Clemente A., Lombó F. (2015). Healthy effects of prebiotics and their metabolites against intestinal diseases and colorectal cancer. AIMS Microbiol..

[74] Kuru-Yaşar R., Üstün-Aytekin Ö. (2024). The Crucial Roles of Diet, Microbiota, and Postbiotics in Colorectal Cancer. Curr. Nutr. Rep..

[75] Fung K.Y., Cosgrove L., Lockett T., Head R., Topping D.L. (2012). A review of the potential mechanisms for the lowering of colorectal oncogenesis by butyrate. Br. J. Nutr..

[76] Liu L., Li Y., Zheng X., Huang R., Huang X., Zhao Y., Liu W., Lei Y., Li Q., Zhong Z. (2024). Natural polysaccharides regulate intestinal microbiota for inhibiting colorectal cancer. Heliyon.

[77] Song L., Zhu S., Liu C., Zhang Q., Liang X. (2022). Baicalin induces apoptosis and inhibits migration in CRC via TLR4/NF-κB pathway. J. Food Biochem..

[78] Laurindo L.F., Santos A.R.O.D., Carvalho A.C.A., Bechara M.D., Guiguer E.L., Goulart R.A., Vargas Sinatora R., Araújo A.C., Barbalho S.M. (2023). Phytochemicals and Regulation of NF-κB in Inflammatory Bowel Diseases: An Overview of In Vitro and In Vivo Effects. Metabolites.

[79] Chen J., Zhong H., Huang Z., Chen X., You J., Zou T. (2023). A Critical Review of Kaempferol in Intestinal Health and Diseases. Antioxidants.

[80] Sauruk da Silva K., Carla da Silveira B., Bueno L.R., Malaquias da Silva L.C., da Silva Fonseca L., Fernandes E.S., Maria-Ferreira D. (2021). Beneficial effects of polysaccharides on the epithelial barrier function in intestinal mucositis. Front. Physiol..

[81] Ruan J., Zhang P., Zhang Q., Zhao S., Dang Z., Lu M., Li H., Zhang Y., Wang T. (2023). Colorectal cancer inhibitory properties of polysaccharides and their molecular mechanisms: A review. Int. J. Biol. Macromol..

[82] Zhang H., Zhu S., Shang D., Hamid N., Ma Q., Xiao Y., Ren L., Liu G., Sun A. (2025). Enhancing stability and physiological activity of *Aronia melanocarpa* L. anthocyanin by polysaccharides. J. Sci. Food Agric..

[83] Jang Y., Koh E. (2023). Characterisation and storage stability of aronia anthocyanins encapsulated with combinations of maltodextrin with carboxymethyl cellulose, gum Arabic, and xanthan gum. Food Chem..

[84] Tong Y., Deng H., Kong Y., Tan C., Chen J., Wan M., Wang M., Yan T., Meng X., Li L. (2020). Stability and structural characteristics of amylopectin nanoparticle-binding anthocyanins in *Aronia melanocarpa*. Food Chem..

[85] Tong Y., Ma Y., Kong Y., Deng H., Wan M., Tan C., Wang M., Li L., Meng X. (2021). Pharmacokinetic and excretion study of *Aronia melanocarpa* anthocyanins bound to amylopectin nanoparticles and their main metabolites using high-performance liquid chromatography-tandem mass spectrometry. Food Funct..

[86] Li Y., Xu C., Weng W., Goel A. (2024). Combined treatment with Aronia berry extract and oligomeric proanthocyanidins exhibit a synergistic anticancer efficacy through LMNB1–AKT signaling pathways in colorectal cancer. Mol. Carcinog..

[87] Wei J., Yu W., Hao R., Fan J., Gao J. (2020). Anthocyanins from *Aronia melanocarpa* induce apoptosis in Caco-2 cells through Wnt/β-catenin signaling pathway. Chem. Biodivers..

[88] Vladu A.F., Ficai D., Ene A.G., Ficai A. (2022). Combination therapy using polyphenols: An efficient way to improve antitumoral activity and reduce resistance. Int. J. Mol. Sci..

[89] Bermúdez-Soto M.J., Larrosa M., Garcia-Cantalejo J.M., Espín J.C., Tomás-Barberán F.A., García-Conesa M.T. (2007). Up-regulation of tumor suppressor carcinoembryonic antigen-related cell adhesion molecule 1 in human colon cancer Caco-2 cells following repetitive exposure to dietary levels of a polyphenol-rich chokeberry juice. J. Nutr. Biochem..

[90] Cvetanović A., Zengin G., Zeković Z., Švarc-Gajić J., Ražić S., Damjanović A., Mašković P., Mitić M. (2018). Comparative in vitro studies of the biological potential and chemical composition of stems, leaves and berries *Aronia melanocarpa* extracts obtained by subcritical water extraction. Food Chem. Toxicol..

[91] Stanca L., Bilteanu L., Bujor O.C., Ion V.A., Petre A.C., Bădulescu L., Geicu O.I., Pisoschi A.M., Șerban A.I., Ghimpeteanu O.M. (2024). Development of functional foods: A comparative study on the polyphenols and anthocyanins content in chokeberry and blueberry pomace extracts and their antitumor properties. Foods.

[92] Valcheva-Kuzmanova S., Marazova K., Krasnaliev I., Galunska B., Borisova P., Belcheva A. (2005). Effect of *Aronia melanocarpa* fruit juice on indomethacin-induced gastric mucosal damage and oxidative stress in rats. Exp. Toxicol. Pathol..

[93] Valcheva-Kuzmanova S., Kuzmanov K., Tancheva S., Belcheva A. (2007). Hypoglycemic and hypolipidemic effects of *Aronia melanocarpa* fruit juice in streptozotocin-induced diabetic rats. Methods Find Exp. Clin. Pharmacol..

[94] Valcheva-Kuzmanova S., Kuzmanov A., Kuzmanova V., Tzaneva M. (2018). *Aronia melanocarpa* fruit juice ameliorates the symptoms of inflammatory bowel disease in TNBS-induced colitis in rats. Food Chem. Toxicol..

[95] Wang S., Wu P., Fan Z., He X., Liu J., Li M., Chen F. (2023). Dandelion polysaccharide treatment protects against dextran sodium sulfate-induced colitis by suppressing NF-κB/NLRP3 inflammasome-mediated inflammation and activating Nrf2 in mouse colon. Food Sci. Nutr..

[96] Zhu Y., Cai P.-J., Dai H.-C., Xiao Y.-H., Jia C.-L., Sun A.-D. (2023). Black chokeberry (*Aronia melanocarpa* L.) polyphenols attenuate obesity-induced colonic inflammation by regulating gut microbiota and the TLR4/NF-κB signaling pathway in high fat diet-fed rats. Food Funct..

[97] Ren Z., Fang H., Zhang J., Wang R., Xiao W., Zheng K., Yu H., Zhao Y. (2022). Dietary *Aronia melanocarpa* Pomace Supplementation Enhances the Expression of ZO-1 and Occludin and Promotes Intestinal Development in Pigs. Front. Vet. Sci..

[98] Pei R., Liu J., Martin D.A., Valdez J.C., Jeffety J., Barrett-Wilt G.A., Liu Z., Bolling B.W. (2019). Aronia berry supplementation mitigates inflammation in T-cell transfer-induced colitis by decreasing oxidative stress. Nutrients.

[99] Naruszewicz M., Łaniewska H., Millo B., Dłużniewski M. (2007). Combination therapy of statin with flavonoids rich extract from chokeberry fruits enhanced reduction in cardiovascular risk markers in patients after myocardial infarction (MI). Atherosclerosis.

[100] Broncel M., Kozirog M., Duchnowicz P., Koter-Michalak M., Chojnowska-Jezierska J., Sikora J. (2010). Aronia melanocarpa extract reduces blood pressure, serum endothelin, lipid, and oxidative stress marker levels in patients with metabolic syndrome. Med. Sci. Monit..

[101] Kardum N., Konić-Ristić A., Savikin K., Spasić S., Stefanović A., Ivanišević J., Miljković M. (2014). Effects of polyphenol-rich chokeberry juice on antioxidant/pro-oxidant status in healthy subjects. J. Med. Food.

[102] Kardum N., Milovanović B., Šavikin K., Zdunić G., Mutavdžin S., Gligorijević T., Spasić S. (2015). Beneficial effects of polyphenol-rich chokeberry juice consumption on blood pressure level and lipid status in hypertensive subjects. J. Med. Food.

[103] Milutinović M., Veličković Radovanović R., Šavikin K., Radenković S., Arvandi M., Pešić M., Kostić M., Miladinović B., Branković S., Kitić D. (2019). Chokeberry juice supplementation in type 2 diabetic patients—Impact on health status. J. Appl. Biomed..

[104] Tasić N., Jakovljevic V.L.J., Mitrovic M., Djindjic B., Tasic D., Dragisic D., Citakovic Z., Kovacevic Z., Radoman K., Zivkovic V. (2021). Black chokeberry *Aronia melanocarpa* extract reduces blood pressure, glycemia and lipid profile in patients with metabolic syndrome: A prospective controlled trial. Mol. Cell. Biochem..

[105] Gancheva S., Ivanova I., Atanassova A., Gancheva Tomova D., Eftimov M., Moneva K., Zhelyazkova Savova M., Valcheva Kuzmanova S. (2021). Effects of *Aronia melanocarpa* fruit juice on oxidative stress, energy homeostasis, and liver function in overweight and healthy-weight individuals. Scripta Sci. Med..

[106] Milosavljevic I., Jakovljevic V., Petrovic D., Draginic N., Jeremic J., Mitrovic M., Zivkovic V., Srejovic I., Stojic V., Bolevich S. (2021). Standardized *Aronia melanocarpa* extract regulates redox status in patients receiving hemodialysis with anemia. Mol. Cell. Biochem..

[107] Stankiewicz B., Cieślicka M., Kujawski S., Piskorska E., Kowalik T., Korycka J., Skarpańska-Stejnborn A. (2021). Effects of antioxidant supplementation on oxidative stress balance in young footballers—A randomized double-blind trial. J. Int. Soc. Sports Nutr..

[108] Stankiewicz B., Cieślicka M., Mieszkowski J., Kochanowicz A., Niespodziński B., Szwarc A., Waldziński T., Reczkowicz J., Piskorska E., Petr M. (2023). Effect of supplementation with black chokeberry (*Aronia melanocarpa*) extract on inflammatory status and selected markers of iron metabolism in young football players: A randomized double-blind trial. Nutrients.

[109] Chung J.W., Kim J.E., Nam Y.E., Kim W.S., Lee I., Yim S.V., Kwon O. (2023). Eight-week supplementation of *Aronia* berry extract promoted the glutathione defence system against acute aerobic exercise-induced oxidative load immediately and 30 min post-exercise in healthy adults: A double-blind, randomised, controlled trial. J. Hum. Nutr. Diet..

[110] Sangild J., Faldborg A., Schousboe C., Fedder M.D.K., Christensen L.P., Lausdahl A.K., Arnspang E.C., Gregersen S., Jakobsen H.B., Knudsen U.B. (2023). Effects of chokeberries (*Aronia* spp.) on cytoprotective and cardiometabolic markers and semen quality in 109 mildly hypercholesterolemic Danish men: A prospective, double-blinded, randomized, crossover trial. J. Clin. Med..

[111] Xu T., Zhang X., Liu Y., Wang H., Luo J., Luo Y., An P. (2021). Effects of dietary polyphenol supplementation on iron status and erythropoiesis: A systematic review and meta-analysis of randomized controlled trials. Am. J. Clin. Nutr..

[112] Eisinaitė V., Leskauskaitė D., Pukalskienė M., Venskutonis P.R. (2020). Freeze-drying of black chokeberry pomace extract–loaded double emulsions to obtain dispersible powders. J. Food Sci..

[113] Buerkli S., Salvioni L., Koller N., Zeder C., Teles M.J., Porto G., Habermann J.H., Dubach I.L., Vallelian F., Frey B.M. (2022). The effect of a natural polyphenol supplement on iron absorption in adults with hereditary hemochromatosis. Eur. J. Nutr..

[114] Bushmeleva K., Vyshtakalyuk A., Terenzhev D., Belov T., Kazimova K., Zobov V. (2025). Effects of *Aronia melanocarpa* tannins on oxidative stress and immune dysfunction. Molecules.

[115] Farhan M., Rizvi A. (2022). Understanding the prooxidant action of plant polyphenols in the cellular microenvironment of malignant cells: Role of copper and therapeutic implications. Front. Pharmacol..

[116] Eghbaliferiz S., Iranshahi M. (2016). Prooxidant activity of polyphenols, flavonoids, anthocyanins and carotenoids: Updated review of mechanisms and catalyzing metals. Phytother. Res..

[117] Nowak M., Tryniszewski W., Sarniak A., Włodarczyk A., Nowak P.J., Nowak D. (2022). Concentration dependence of anti- and pro-oxidant activity of polyphenols as evaluated with a light-emitting Fe^2+^-EGTA-H_2_O_2_ system. Molecules.

[118] Lambert J.D., Sang S., Lu A.Y., Yang C.S. (2007). Metabolism of dietary polyphenols and possible interactions with drugs. Curr. Drug Metab..

[119] Lackner S., Li Y., Timm J., Siegmund B., Smollich M., Bork P., Haller D. (2024). Interindividual differences in aronia juice tolerability linked to gut microbiome and metabolome changes—Secondary analysis of a randomized placebo-controlled parallel intervention trial. Microbiome.

